# Supplementary material for: Distinct neutralization sensitivity between adult and infant transmitted/founder HIV-1 subtype C viruses to broadly neutralizing monoclonal antibodies
Source: PLoS Pathog. 2025 Jun 23;21(6):e1013245. doi: 10.1371/journal.ppat.1013245 (PMC12225807; doi:10.1371/journal.ppat.1013245)
Supplement: S1 Table — FRESH PID = Participant Identifier; Intrapatient diversity score calculated as the average pairwise nucleotide difference; % diversity = intrapatient diversity score expressed as a percentage. (DOCX) [file ppat.1013245.s001.docx]

| **FRESH PID** | | **Intrapatient diversity score** | **% diversity** |
| --- | --- | --- | --- |
| 1 | 498 | 0,017 | 1,7 |
| 2 | O79 | 0,001 | 0,1 |
| 3 | 857 | 0,005 | 0,5 |
| 4 | 102 | 0,000 | 0,0 |
| 5 | 1439 | 0,000 | 0,0 |
| 6 | 318 | 0,000 | 0,0 |
| 7 | O36 | 0,000 | 0,0 |
| 8 | 651 | 0,001 | 0,1 |
| 9 | 186 | 0,000 | 0,0 |
| 10 | 970 | 0,000 | 0,0 |
| 11 | 1074 | 0,001 | 0,1 |
| 12 | 519 | 0,000 | 0,0 |
| 13 | 272 | 0,000 | 0,0 |
| 14 | O39 | 0,000 | 0,0 |
| 15 | 2148 | 0,004 | 0,4 |
| 16 | 1512 | 0,000 | 0,0 |
| 17 | 594 | 0,001 | 0,1 |
| 18 | O93 | 0,000 | 0,0 |
| 19 | 198 | 0,000 | 0,0 |
| 20 | 1388 | 0,003 | 0,3 |
| 21 | 726 | 0,001 | 0,1 |
| 22 | 201 | 0,019 | 1,9 |
| 23 | 479 | 0,004 | 0,4 |
| 24 | 1088 | 0,000 | 0,0 |
| 25 | 1952 | 0,005 | 0,5 |
| 26 | 451 | 0,008 | 0,8 |
| 27 | 267 | 0,002 | 0,2 |
| 28 | 268 | 0,000 | 0,0 |
| 29 | 922 | 0,020 | 2,0 |
| 30 | 559 | 0,001 | 0,1 |
| 31 | 627 | 0,008 | 0,8 |
| 32 | 208 | 0,000 | 0,0 |
| 33 | 1368 | 0,000 | 0,0 |
| 34 | 920 | 0,005 | 0,5 |
| 35 | 271 | 0,017 | 1,7 |
| 36 | 499 | 0,000 | 0,0 |
| 37 | 527 | 0,002 | 0,2 |
| **Median** | | | **0,1** |

**Supp Table 1**
