# Supplementary figures and images for: Distinct neutralization sensitivity between adult and infant transmitted/founder HIV-1 subtype C viruses to broadly neutralizing monoclonal antibodies
Source: PLoS Pathog. 2025 Jun 23;21(6):e1013245. doi: 10.1371/journal.ppat.1013245 (PMC12225807; doi:10.1371/journal.ppat.1013245)

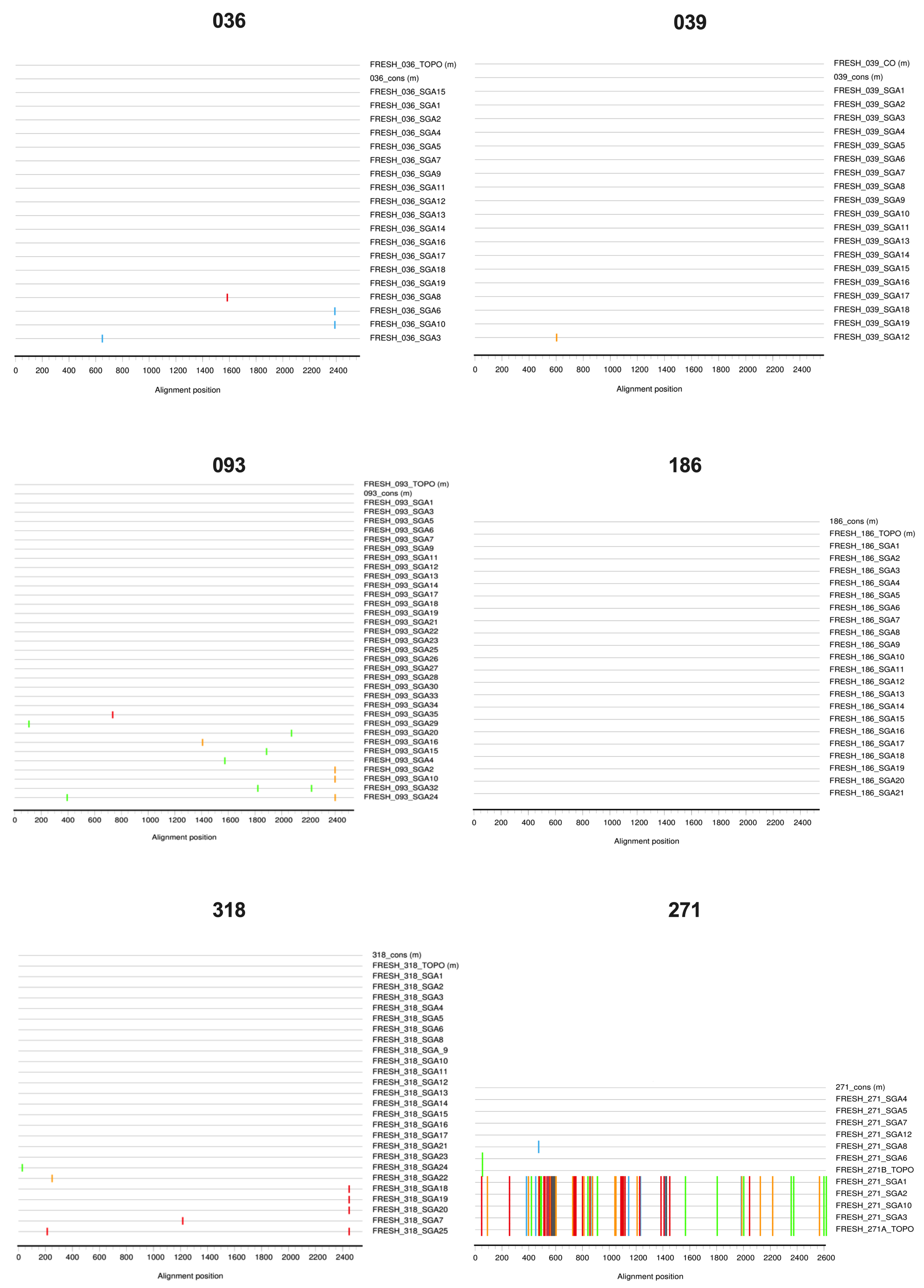

Supplement: S1 Fig — Representative Highlighter plots were selected to illustrate typical levels of intrapatient diversity observed among FRESH participants. Each horizontal line represents a single viral sequence derived from HIV-1 env single genome amplification (SGA) for individual participants. Colored vertical ticks indicate the positions and types of nucleotide substitutions relative to the participant-specific consensus sequence and the representative env clone used for neutralisation assays. Substitution types are color-coded as follows: A → G (green), G → A (yellow), C → T (red), T → C (light blue), transversions (other colors). (TIFF) [file ppat.1013245.s002.tiff]
