# Supplementary material for: Distinct neutralization sensitivity between adult and infant transmitted/founder HIV-1 subtype C viruses to broadly neutralizing monoclonal antibodies
Source: PLoS Pathog. 2025 Jun 23;21(6):e1013245. doi: 10.1371/journal.ppat.1013245 (PMC12225807; doi:10.1371/journal.ppat.1013245)
Supplement: Attachment — Submitted filename: Response to Reviewers_07 May 2025.docx [file ppat.1013245.s005.docx]

**May 07, 2025**

The Editors, *PLoS Pathogens*

### Dear Editors,

### **RE: Manuscript Ref. No. PPATHOGENS-D-25-00262 - [EMID:03b56fb494d9da0f]**

Please find attached a copy of the revised manuscript entitled “**Distinct neutralization sensitivity between adult and infant transmitted/founder HIV-1 subtype C viruses to broadly neutralizing monoclonal antibodies.**” We thank you very much for the opportunity to revise our manuscript. We have carefully considered the editors and reviewers comments and suggestions, and where appropriate, we have modified our manuscript in response to these helpful comments. We believe that the changes we have made to the manuscript add clarity to the data and strengthen the manuscript. We have addressed specific comments as follows (reviewers comments are in italics):

**Part I: General Comments**

1. *Many published studies characterize viruses from repository samples, which may be years to decades old. The samples used in these studies are taken from ongoing cohorts but it is unclear as to whether the samples are truly “contemporaneous”. When were the samples used in these studies obtained?*

Response: We thank the reviewers for the useful comments on our manuscript. This study characterized viruses that represent samples from ongoing cohort studies that were available to us at the time (2013-2020) for the FRESH cohort and 2015-2020 for the Baby Cure study. We agree with the reviewer that the samples are not exactly contemporaneous, this was revised throughout the document. The period of sample collection was added to the Study Population section (lines 158-161). We have ongoing studies focusing on virus evolution that are characterizing contemporaneous viruses (acute infection viruses from within the last 3 years) and comparing them to historical viruses (more than a decade old).

2. *For these studies, HIV was amplified from plasma obtained from a prospective cohort of women who were monitored twice weekly using nucleic acid testing for primary infection. Samples used in these studies were taken within 7 days (median 1 day) following first detection of nucleic acids. Of note, almost half of the women were Fiebig 2-4 at testing and many of these women had extremely high viral loads. Was either PacBio or Illumina sequencing done on the FRESH samples? A median of only 6 SGA env were generated for the study; it would be interesting to see how representative the SGA env used were in the assays were of the viral population present at the time of sampling.*

Response: PacBio or Illumina sequencing was only performed for the transmitting mothers in the Baby Cure Cohort. Bulk amplification and Sanger sequencing were performed for infants, due to technical difficulties and sample volume limitations. In addition, sequences in the FRESH cohort were generated by Sanger sequencing after a limiting dilution single genome amplification approach. Although a median of only 6 env SGAs per participant were generated, we can be confident that the env clones are representative of the viral population at the time of sampling because phylogenetic analysis revealed highly homogenous sequences, with minimal diversity. In the cases of distinct clusters, env clones from each cluster where selected for neutralization analysis. SGA amplicons were used to calculate intrapatient nucleotide diversity in 38 participants (Supp Table 1), and most of them had low diversity scores (<1%). Seven participants showed noticeable nucleotide diversity ranging from 1-2% (line 383-387). Highlighter plots were also constructed as illustrated for representative samples in Supp Fig 1, and most participants had very limited nucleotide sequence variation, consistent with low intrapatient sequence diversity. In the seven participants where SGA sequence analysis revealed distinct phylogenetic clusters, representative clones from these clusters were selected for functional analysis on bnAb sensitivity. For example, for participant 271, two representative SGAs were selected (271A and 271B) from phylogenetically distinct clusters. We are less confident about how representative the infant samples are because we used bulk amplification and sequencing but previous studies show that similar to heterosexual transmission, there is a genetic bottleneck during mother to child transmission with transmitted/founder viruses being highly homogenous. However, we have also acknowledged the limitation of bulk amplification for the infant samples in the Discussion section (lines 743-746).

3. *The majority (16) of infant samples used in these studies were taken within 48 hours of birth, while a few (4) were obtained at one month. It would be helpful in Table 1 to distinguish the timing of samples designated as obtained at birth. As well as the prophylactic ART (NVP+-ZDV) that the infants received. SGA’s were not done due to limited plasma sample volumes and only a single env clone was generated and tested in most infants yet from Table 2, it looks like the viral loads in most samples were high enough to allow SGA using very small amounts of plasma.*

Response: We thank the reviewer for this helpful suggestion. We have revised Table 2 accordingly, to distinguish the timing of sample collection, and the prophylactic ART regimen is now included as a footnote to table 2. Single genome amplification was attempted for the infant plasma samples, however most of the SGA amplification failed. While we are not sure of the reasons for the failed amplification, we did not have enough samples to repeat the SGA amplifications, and therefore performed HIV-1 bulk PCR amplification. It should be noted that blood samples from infants were small in volume and had to be split between this study and clinical assays that needed to be prioritized for optimal clinical management of HIV. We acknowledge the limitation that bulk PCR amplification may not fully represent the diversity of quasispecies within a sample but this is somewhat mitigated by the limited diversity of transmitted/founder viruses due to the well-described genetic bottleneck that occurs at transmission. Therefore, these Env sequence data are relevant for the selection of bnAbs in paediatric settings. This was listed as one of the study limitations (lines 724-729)

4. *As the authors point out, only a few matched maternal-infant pairs were characterized in this study, which seems like a missed opportunity to examine mechanisms of vertical transmission and the reported preferential selection of escape mutants.*

Response: We agree with the reviewer that there is a lot to be gained from studies of sequences of mother-infant pairs from this cohort. However, our main goal for this study was to determine the characteristics of the transmitted/founder viruses in infants and analysis of mother-infant pairs is outside the scope of the current study. The differences observed between adults and infants suggested different mechanisms of adult versus infant HIV transmission, which then led us to characterize a few mother-infant pairs as part of this study. We believe that the ongoing comprehensive study is better suited to provide valuable information on the mechanisms of vertical transmission and preferential selection of escape mutants.

5. *The study focuses on bnAb neutralization. How might other effector functions described for some of the bnAbs (e.g. Fc-receptor mediated) contribute to immune clearance even when reduced neutralization is observed.*

We agree with the reviewer that this study only focused on the ability of bnAbs to neutralize subtype C viruses. We do not know whether these bnAbs also elicited Fc-mediated effector functions, and now mention this as one of the limitations of this study (lines 736-739).

**Part II Major Issues: Key Experiments Required for Acceptance**

**Reviewer 1:**

1. *What is the evidence that the viruses from the FRESH and Baby Cure cohorts are confidently TF viruses? Is it solely based on time of virus extraction, ie within 48 hours after birth in babies and median of 1-day post plasma viremia in adults? It would be helpful for the authors to outline the criteria for defining TF viruses in the study; perhaps a section in the methods where readers can access this information and make their own conclusions based on this information. If there are uncertainties in classification of these viruses, the authors should also outline them in a new section of the methods (this is homehow addressed in lined 607-613 but could be more fleshed out in the methods.*

Response: We acknowledge that there may be some uncertainty on the definition of transmitted/founder viruses. The evidence we have that the viruses we sequenced are transmitted/founder viruses is based on the well-characterized timing of infection and/or first detection of plasma viremia in both the FRESH and Baby Cure cohorts. In the FRESH cohort in particular, there was evidence of a previous HIV-1 negative test of plasma, usually within 4 days. Viral sequences generated by a limiting dilution approach (single genome amplification) were also highly homogeneous, consistent with the well-described pattern of a genetic bottleneck in HIV-1 transmission where only a few or very limited number of sequences establish an infected in the newly infected host. In the case of infants, transmission could have occurred in utero, and therefore the exact timing relative to the viral sequences generated is unknown. We acknowledge this limitation and have added a description of our criteria of defining the transmitted/founder viruses in the methods section (line 248-268).

2. *It would be more helpful to the readers if the authors revised figures 3 and 4 to include virus names, neutralization tiers (1/2/unknown) and clade/subtype. The PIDs or numbers shown are not informative. Additionally, there is an emerging discussion in the HIV field of whether the current panels of HIV strains used to assess breadth of bnAbs from adults are appropriate to assess bnAbs from pediatric populations. There are two bnAbs previously isolated from pediatric populations, BF520 and AIMS_330; both of which were tested on different virus panels for breadth. There is an opportunity here for the authors to test BF520 and AIMS_330 against the adult and infant TF viruses in this study as it would be helpful to the field to see whether the sensitivity of adult or infant TF viruses show differences in sensitivity to infant bnAbs. These results may provide insights into whether current bnAbs from adults are adequate for treating infants living with HIV.*

We thank the reviewer for this useful suggestion, Figure 3A and 3B were revised to include the virus subtypes. However, the full virus/participant identification numbers were not included to maintain confidentiality, as these are patient identification numbers. We also welcome the suggestion of testing BF520 and AIMS-330 but we were unable to get access of these bnAbs through the NIH’s AIDS Reagent Program. We plan to pursue this suggestion for a follow up study that will also include other isolated monoclonals and sera from children with cross-reactive neutralization activity.

3. *To better understand the results in lines 392-395, is there any evidence that the structures of Envs from TF viruses in infants have modifications or differences (CD4-binding site) from those in adults, thus influencing the enhanced sensitivity to majority of the bnAbs by the baby TF viruses compared to those from adults? Maybe something for the authors to also speculate on based on information that can be ascertained from the viral Env sequencing if structural analysis is not possible.*

Response: We thank the reviewers for this great suggestion, we agree that structural analysis may provide valuable information in the differences between adults and infants envelopes and their interactions with various bnAbs. In response to this comment, we have added some speculations on the structure, accessibility and N-linked glycosylation patterns in lines 459-464.

4. *The authors did a great job with sequencing analyses to inform bnAb sensitivity or resistance, but some key residues or regions were not mentioned and it is not clear whether these analyses were done and the results not informative or whether they were excluded. For example, the residues in the loop D, V5 loop and bridging sheet were studied, but not the CD4-binding loop; all of which comprises the CD4-binding site. Any evidence for conserved residues in the CD4-binding loop such as D368 or mutations at this site that would influence sensitivity or resistance to CD4bs antibodies. For V2 apex, no information provided on N156 in the V2 apex. For V3 glycan sensitivity, it is becoming more clearer that Envs with short V1 or V1 glycan deletions are more sensitive to V3 glycan bnAbs due to access to the V3 glycan bnAb epitope, so these may be other areas to study to better understand sensitivity to V3 glycan bnAb epitope. Finally, in MPER, only distal MPER epitopes assessed that is relevant for 10E8 targeting, but proximal MPER Abs have been recently generated via vaccination in adult humans and SIV-infected macaques, so any information on sequence variability in this region may be helpful to fully understand how MPER bnAbs can be incorporated into neonatal responses to HIV.*

Response: We thank the reviewers for these useful comments. Position D368 of the CD4 binding loop and N156 glycans of the V2 apex were added in the LogoSeq Plots (Fig 6) and variations in amino acid signatures were compared between adults and infants. Aspartic acid (D) at position 368 was highly conserved, while minor variations were found in position 365 in both FRESH and Baby Cure cohorts (line 534-539). In addition, minor variations were found at position N156, most of the escape mutations were observed in FRESH than Baby Cure sequences (line 550-559). The V1 loop length and potential N-linked glycosylation sites were compared between FRESH and Baby Cure and there were no significant differences, although few variants in each group had very long V1 loops and they were associated with resistance to V3 glycan bnAbs (line 595-603). The proximal region of MPER including amino acids in position 662-668 that form the target epitopes for 2F5 bnAb were also analyzed. There was a high frequency of escape mutations in the proximal MPER region, while the distal MPER was highly conserved in both cohorts (line 605-631), suggesting that subtype C vaccine efforts should prioritize the distal 10E8 epitope.

5. *For such a high impact study, the discussion section could be revised to contribute to ongoing discussions in the field of pediatric HIV immunity. First lines 553-605 in some part were already reported in the results section and could be significantly reduced to make the main points of sensitivity to different bnAbs. Second, as the authors appropriately mentioned studying the moms in the Baby Cure cohort could provide insights into the potential mechanisms of viral transmission/ dynamics of virus sensitivity to bnAbs from adults to infants. If virus is more resistant to bnAbs in adults but more sensitive to said bnAbs and only the bnAb-sensitive one get transmitted or is there some biological factors at play to alter virus bnAb sensitivity in utero or soon after birth? The authors could speculate on some of these concepts in the discussion. Third, these results may provide implications for HIV vaccine designs in pediatric populations. For instance, do the authors see any value in these Envs with sensitivity to bnAbs serving as appropriate immunogens in future HIV vaccine strategies? These are all areas of interest to the field that could be incorporated into the discussion section.*

Response: We appreciate and acknowledge these insightful comments. We have revised the discussion section accordingly to summarize the description of results (line 657-672) and addressed some of the points raised by the reviewers (line 685-718) and made changes on the limitations of the study (line 724-739).

**Reviewer 2: N/A**

**Reviewer 3:**

*One concern is how representative the env used for these studies are of T/F viruses. For example, a median of only 6 SGA env were generated for the study of FRESH cohort samples; it would be interesting to see how representative the SGA env used in the assays were of the viral population present at the time of sampling. Similarly, single infant env derived from bulk PCR were evaluated despite high viral loads in many infants.*

We acknowledge the limitation of the Sanger sequencing technique that was used in this study. However, the intrapatient diversity was calculated in the FRESH cohort and most of the participants had low intrapatient diversity. Highlighter plots also show low frequency of sequence diversity in most of the participants. However, in seven participants that had higher diversity, we selected more than one variant to represent each population of variants. Please refer to the general comments.

**Part III: Minor Issues: Editorial and Data Presentation Modifications**

**Reviewer 1: Additional minor concerns:**

1. *Update language throughout the manuscript from HIV-1 infected to people living with HIV e.g. lines 136, 138, 273, 276 ect*

The language was updated accordingly, HIV infected people was changed to people living with HIV in lines 158, 161, 326, 330

2. *To avoid confusion, whenever the authors reference mothers, they should consider indicating mothers for infants in the Baby Cure cohort, for e. lines 286-287.*

“Mothers” was changed to “mothers for infants in the Baby Cure cohort” on line 338-345

3. *Treatment regimen at bottom of table 2; how does this link to the table? More information is needed here, including symbols?*

The treatment regimen for mothers and infants were clarified in footnotes below table 2. Infants received the combination of AZT (Zidovudine), Lamivudine (3TC) and Nevarapine (NVP). While mothers received the combination of Efavirenz (EFV), Tenofovir disoproxil fumerate (TDF) and Emtricitabine (FTC).

4. *Figure 1 legend should indicate timepoints of samples for viral load measurements and length of time on ART. This information would provide clarity to the readers when reviewing these data.*

Timepoints of sample collection for viral load and CD4 counts measurements were included in the figure legend (line 361-364).

**Reviewer 2:** *Stats in Fig 1 don’t include infants overall and mom overall? Were the medians tested and found non-significant?*

The statistical analysis was performed on the overall infants and their mothers VL and the figure was added as (Fig 1C). Infants had higher VL compared to their transmitting mothers (p= 0.02).

*Fig 1B and C is mistakenly described as Fig 2B and C in the results section. Not super clear in the figure legend what the difference is between Fig 1A and 1B or why transmitting mothers would be different from FRESH cohort?*

Corrections were made, Fig 2B and C were changed to Fig 1B and C in the results section, Fig 1A compares viral load between FRESH participants and infants in the Baby Cure cohort, while Fig 1B compares viral load between FRESH participants and transmitting mothers from the Baby Cure cohort. These are different cohorts. Plasma viral loads are significantly different because transmitting mothers were on antiretroviral therapy, while the FRESH participants were newly infected with HIV (line 338-345), samples were collected before treatment initiation.

We thank you again for the opportunity to revise our manuscript and are grateful to the reviewers for their helpful and constructive comments and suggestions. We believe that this work is now greatly improved and hope it will be found acceptable for publication in *PLoS Pathogens*.

Yours Sincerely,

**Thumbi Ndung'u, BVM, PhD**

Professor and Director for Basic and Translational Science

Africa Health Research Institute and

HIV Pathogenesis Programme, University of KwaZulu-Natal
